# The Morphometry of Male Genitalia as a Reliable Tool for Identifying Forest Pests *Dendrolimus sibiricus*, *D. pini* (Lepidoptera: Lasiocampidae), and Their Hybrids in Eurasia

**DOI:** 10.3390/life16030398

**Published:** 2026-03-01

**Authors:** Maria A. Ryazanova, Alexander A. Ageev, Sergey Yu. Sinev, Alexey Yu. Matov, Stanislav Gomboc, Margarita G. Kovalenko, Evgeny N. Akulov, Denis A. Demidko, Dmitrii L. Musolin, Natalia I. Kirichenko

**Affiliations:** 1 Sukachev Institute of Forest, Siberian Branch of the Russian Academy of Sciences, Federal Research Center “Krasnoyarsk Science Center SB RAS”, Krasnoyarsk 660036, Russia; sawer_beetle@mail.ru; 2 Institute of Ecology and Geography, Siberian Federal University, Krasnoyarsk 660041, Russia; 3 All-Russian Research Institute of Forestry and Forestry Mechanization (VNIILM), “Forest Pyrology Center”, Krasnoyarsk Branch, Krupskoy St. 42, Krasnoyarsk 660062, Russia; ageevaa@firescience.ru; 4 Zoological Institute of Russian Academy of Sciences, 1 Universitetskaya Emb., St. Petersburg 199034, Russia; sinev@zin.ru (S.Y.S.); noctua2006@yandex.ru (A.Y.M.); 5 Independent Researcher, Gančani 110, 9231 Beltinci, Slovenia; stanislav.gomboc@siol.net; 6 All-Russian Plant Quarantine Center (VNIIKR), Pogranichnaya Str. 32, Moscow 140150, Russia; bush_zbs@mail.ru; 7 Krasnoyarsk Branch of Centre for Agriproducts Quality Assurance, Zhelyabova Str. 6/6, Krasnoyarsk 660020, Russia; akulich80@yandex.ru; 8 European and Mediterranean Plant Protection Organization, 21 Boulevard Richard Lenoir, 75011 Paris, France; musolin@gmail.com

**Keywords:** Siberian moth, pine-tree lappet, hybridization, genital morphometry, genitalia indices, species identification, quarantine diagnostics

## Abstract

The Siberian moth, *Dendrolimus sibiricus* Tschetverikov, is one of the most destructive conifer pests in Northern Asia, causing severe ecological and economic losses. In Russia, its range overlaps with that of the closely related pine-tree lappet *Dendrolimus pini* (L.), and this raises the potential for hybridization and complicates accurate identification, particularly in the context of the potential westward expansion of *D. sibiricus.* Here, we present the first comprehensive morphometric analysis of male genitalia aimed at distinguishing these two major forest pests and their hybrids. The study was based on *D. sibiricus* and *D. pini* specimens collected during the last 130 years (1894–2024) across Europe and Asia, including their hybrids reared indoors by crossing *D. pini* females with *D. sibiricus* males in 1956 and preserved in the collection of the Zoological Institute of the Russian Academy of Sciences (St. Petersburg, Russia). Overall, 70 permanent genitalia slides were prepared (33 *D. sibiricus*, 33 *D. pini*, and 4 hybrids), and the following genital structures were measured: valva and harpe length, aedeagus width and length, and cornuti length. *Dendrolimus sibiricus* had significantly larger genital structures compared to *D. pini*: 74% longer harpe, 32% longer valva, and a 28% wider and longer aedeagus. In contrast, in *D. sibiricus* cornuti were 21% shorter than in *D. pini.* Hybrids displayed intermediate values for valva, harpe, and aedeagus lengths, and for these parameters, they significantly differed from both parental species. The following diagnostic indices were suggested to distinguish between the two species and their hybrids: Harpe Length/Valva Length Index (HL/VL) and Cornuti Length/Aedeagus Length Index (CL/AL). Decision-tree analysis identified HL/VL as the strongest predictor for separating the parental species and the Combined Genital Proportion Index (CGPI), which integrates harpe, valva, aedeagus, and cornuti lengths, as the strongest predictor for identifying hybrids. The morphometric criteria developed here have practical applications for monitoring programs and quarantine diagnostics, particularly in sympatric zones and regions at risk of *D. sibiricus* expansion.

## 1. Introduction

The Siberian moth, *Dendrolimus sibiricus* Tschetverikov, 1908 (Lepidoptera: Lasiocampidae), is one of the most destructive defoliators of coniferous forests in Northern Asia [1,2]. The outbreaks of this pest affect thousands of hectares of taiga, resulting in profound ecological and economic losses [1,3,4,5,6]. Due to its invasive potential and severe impact, *D. sibiricus* is considered a quarantine pest species by the European and Mediterranean Plant Protection Organization (EPPO) and the Eurasian Economic Union (EAEU) [7,8]. Larvae of *D. sibiricus* develop on *Abies*, *Larix*, *Picea*, and *Pinus* species (Pinaceae) [3,9], among which the two-needle pine *Pinus sylvestris* is a poor host [10,11,12].

The native range of *D. sibiricus* spans much of Russia, northern parts of Kazakhstan, Mongolia, China, and Korea [3,9,13,14,15]. In Russia, the species is present in its Asian part (Siberia and the Russian Far East), the Urals and in some regions of the European part of the country [3,15]. Its westernmost distribution boundary in the country remains uncertain [15,16]. Low genetic diversity observed in *D. sibiricus* in European Russia suggests its westward expansion [15].

A closely related species, the pine-tree lappet, *Dendrolimus pini* (Linnaeus, 1758), feeds primarily on two-needle pines, such as *P. sylvestris*. This moth is distributed across most of Europe (from the UK, with a breeding population in Scotland, to Ukraine and from Nordic countries to the Mediterranean region), North Africa (Morocco), Türkiye, Georgia, Russia (the European part and Asian part, including Zabaikalsky Territory, or Transbaikalia), and Kazakhstan [3,17,18,19,20,21,22,23]. In Russia, its range overlaps with that of *D. sibiricus* [3,14,15,20].

External morphology provides little or no reliable tool for distinguishing these two *Dendrolimus* species: forewing pattern and coloration show considerable intraspecific variability in both taxa [3,14]. In females, the features of genitalia are weakly expressed [3,14,24]. Male genitalia, on the contrary, are used to distinguish these two species; however, male genital characters exhibit variation that complicates species identification. DNA barcoding likewise provides limited discriminatory power for differentiating the two species [15,25]. Conflicting identifications likely arise not only from their close phylogenetic relationship but also from species misidentification in molecular genetic public repositories (BOLD and GenBank) [26].

Morphological similarity between *D. sibiricus* and *D. pini* and the co-occurrence of these two species in mixed coniferous forests in Russia suggest a possibility of close interactions in sympatric zones, including potential hybridization [15]. Furthermore, males of *D. pini* are attracted to traps baited with the synthetic sex pheromone of *D. sibiricus* [27,28,29].

Indeed, hybridization within the genus *Dendrolimus* is not merely hypothetical. In a controlled laboratory experiment in China, two other related *Dendrolimus* species, namely, *D. punctatus* (Walker, 1855) and *D. tabulaeformis* Tsai et Liu, 1962, showed the ability to hybridize and produce viable F_1_–F_3_ generations [30]. This finding underlines that reproductive barriers may be evolutionary weak or incomplete in close *Dendrolimus* lineages.

During the revision of *Dendrolimus* moths in the archival collection of the Zoological Institute of the Russian Academy of Sciences (ZIN RAS, Saint Petersburg, Russia)—the country’s largest entomological repository—several individuals labeled as hybrids were discovered, with the inferred parental species and sex indicated. Together with verified specimens of *D. sibiricus* and *D. pini* from the same collection, they provided a unique resource for our study.

In the present study, we investigated male genitalia morphometry to identify diagnostic characters. Based on the hypothesis that some genital structures may exhibit allometric relationships due to their anatomical proximity and functional integration, we introduced reliable morphometric indices for distinguishing the parental species and the hybrids. Establishing diagnostic indices is particularly important for ensuring accurate species identification in both routine diagnostics and pheromone-based monitoring, refining our understanding of the current range of *D. sibiricus* and enabling the timely detection of its possible accidental spread.

## 2. Materials and Methods

### 2.1. Study Region

**Figure 1:**
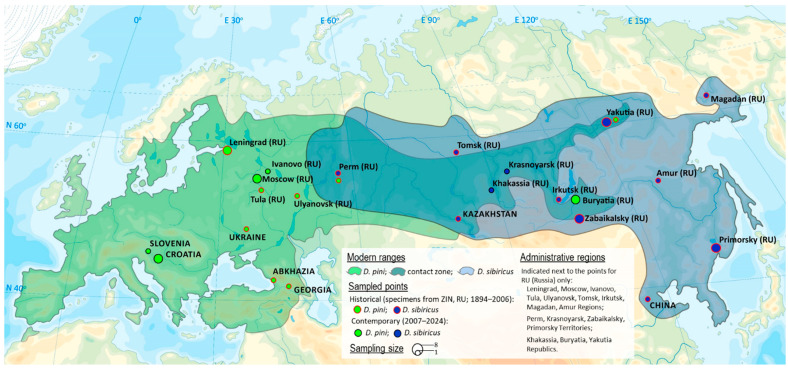
The distribution of *Dendrolimus sibiricus* and *D. pini* with their overlapping zone and sampled points. The map was generated using ArcGIS 9.3 [31].

The study encompassed Europe (within the core range of *D. pini*), the European part of Russia (where the ranges of *D. pini* and *D. sibiricus* partially overlap), and Northern Asia, or the Asian part of Russia (the core range of *D. sibiricus* and the eastern part of the *D. pini* distribution) (Figure 1).

Overall, *Dendrolimus* specimens used for the study originated from Russia (*D. sibiricus* and *D. pini*), Kazakhstan (*D. sibiricus*), Georgia, Abkhazia, Slovenia, Croatia, and Ukraine (*D. pini*). Within Russia, the specimens were from 16 administrative regions spanning the breadth of the country—from its westernmost to easternmost territories: Leningrad, Moscow, Ivanovo, Tula, Ulyanovsk, Tomsk Regions, Perm, Krasnoyarsk Territories, the Republics of Khakassia, Buryatia, Sakha (Yakutia), Irkutsk Region, Zabaikalsky Territory, Magadan, Amur Regions, and Primorsky Territory (Figure 1). This extensive geographic coverage provided a broad representation of both species and their intra- and interspecific variations across their individual and sympatric ranges.

### 2.2. Specimen Sampling

**Figure 2:**
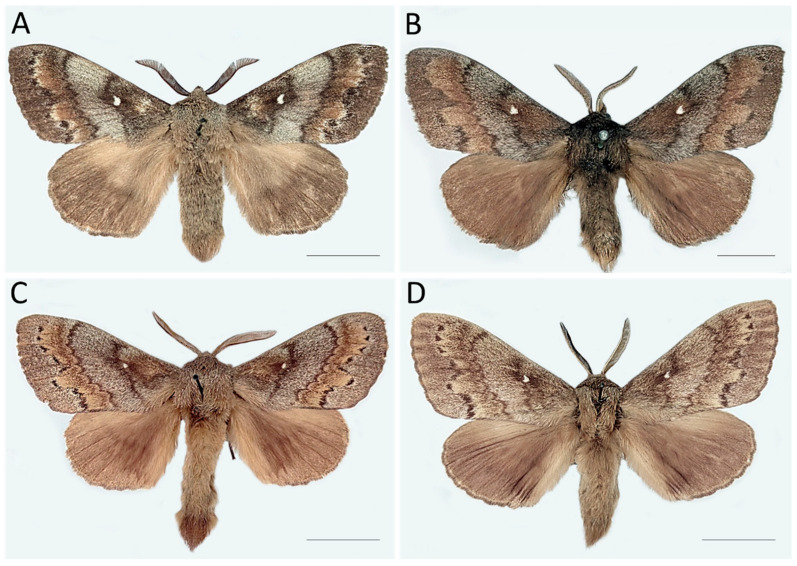
Males of the studied representatives of *Dendrolimus*: (**A**)—*Dendrolimus sibiricus*, (**B**)—*D. pini*, (**C**,**D**)—hybrids. Sampled regions: (**A**)—Russia, Primorsky Territory, Suchansky mine, 19.07.1934, Palshkov coll.; (**B**)—Russia, Tula Region, near Aleksin city, 15.07.1902, Keler coll.; (**C**,**D**)—hybrids (*D. pini* females × *D. sibiricus* males) obtained in an experiment by V.I. Kuznetzov in 1956. All specimens are stored in ZIN RAS. Scale bars = 10 mm. Photos: M.A. Ryazanova and N.I. Kirichenko.

A total of 70 adult male specimens of *Dendrolimus* were examined: 33 *D. sibiricus*, 33 *D. pini*, and 4 hybrid males (Table S1; Figure 2).

The principal material for the study was borrowed from the archival collection of ZIN RAS. This included 44 male specimens (30 *D. sibiricus*, 10 *D. pini*, and 4 hybrids) (Figure 2). Adult *D. sibiricus* and *D. pini* were collected in ten regions of Russia, as well as in Abkhazia, Kazakhstan, and Georgia over 112 years (1894–2006) (Table S1). The hybrids were produced in an experiment crossing *D. pini* females with *D. sibiricus* males by Vladimir I. Kuznetzov in 1956 (Table S1). Unfortunately, no published or archival documentation from this pioneering hybridization experiment is available.

To improve representation across the species’ modern ranges and account for possible geographic variation in genital morphology, 26 specimens collected in 2007–2024 were additionally included in the study (Figure 1). From them, 13 specimens were borrowed from the entomological collections of the All-Russian Plant Quarantine Center (VNIIKR, Bykovo, Moscow Region) and 13 were from private scientific collections (Table S1). These included *D. sibiricus* males captured in pheromone traps in the Krasnoyarsk Territory and the Republic of Khakassia (2023–2024) and *D. pini* males collected in the Moscow and Ivanovo Regions and the Republic of Buryatia (2007–2024), all in traps baited with the synthetic sex pheromone of *D. sibiricus* produced at VNIIKR. Additional *D. pini* males were attracted to light in Slovenia and Croatia (2023–2024).

For all specimens borrowed from ZIN RAS, the terminal third of the abdomen containing male genitalia was carefully cut out using sterilized surgical scissors. After each manipulation, the instrument was rinsed in 95% ethanol and flamed to prevent cross-contamination in the archival collection. The dissected abdomens were placed individually in Eppendorf tubes and transferred to the Forest Zoology Department at the Sukachev Institute of Forest (SIF SB RAS, Krasnoyarsk, Russia), where genital dissection was performed and male genitalia structures measured. Relatively recently collected specimens of *D. sibiricus* and *D. pini* were shipped to the same laboratory, ensuring that all material (historical and contemporary) was processed under the consistent conditions, primarily by the first author.

### 2.3. Preparation of Male Genitalia Slides

A total of 70 permanent male genitalia slides were prepared: 33 slides for *D. pini*, 33 for *D. sibiricus*, and 4 for hybrid specimens. Genital dissections followed the methodology described in [32], with some modifications to improve preparation quality of volumetric and strongly chitinized male genitalia of *Dendrolimus*.

Abdominal segments VI–VIII containing the genitalia were placed in 2 mL tubes filled with 15% KOH and heated in a Dry Block Heater MINIB-100 (Miulab, Hangzhou, China) at 75 °C for 25 min. After maceration, genital apparatus was rinsed in water, and remaining abdominal tissues were removed with a fine brush. Water was replaced one or two times until the genital structures were properly cleaned. During this process, the aedeagus was separated from the genital capsule for subsequent treatment. Within the genital capsule, the symmetrical paired structures (valvae, harpes, and claspers) were spread laterally, which was achieved by a cross-cut through the partially sclerotized intermediate elements—saccus, uncus, tegumen, and juxta.

The cleaned genital capsule and detached aedeagus were then immersed in 50% citric acid for degreasing, followed by lactic acid to clarify and soften the structures. After acid treatment, the preparations were rinsed in 70% ethanol and then transferred to 95% ethanol for dehydrating and hardening. While in 95% ethanol, the paired genital structures, i.e., valvae, harpes, and claspers (or cubiles), were gently flattened into a lateral, two-dimensional plane using a blunt probe. At the same time, the vesica of the aedeagus was everted to allow access to the cornuti situated in two symmetrical groups at the vesica tip.

The flattened genital capsule and the aedeagus with everted vesica were transferred from 95% ethanol onto a glass slide in a drop of Canada balsam. The structure was gently lowered into the balsam to minimize bubble formation and to facilitate the release of residual ethanol. The flattened genital capsule was positioned in the upper two-thirds of the drop and the aedeagus below it. A cover glass was placed above the Canada balsam drop with the genital apparatus and lightly pressed with a blunt probe to ensure even distribution of the balsam and the absence of empty spaces along the edges. As a result, the genitalia slides were properly flattened and unobstructed by residual tissues, balsam artifacts, or overlapping elements of the genital capsule.

The slides were labeled and left horizontally at a temperature of 25 °C for about four months until the balsam fully polymerized. After curing, they were stored vertically in slide boxes. Overall, 20 genitalia slides were deposited at ZIN (Saint Petersburg, Russia), and the other 50 slides were stored in SIF SB RAS (Krasnoyarsk, Russia).

### 2.4. Measurements of Genitalia Structures

The genitalia slides were used for morphometric measurements. Prior to the measurements, the genitalia structures were photographed using binocular stereomicroscope Zeiss Stemi DV4 (Zeiss, Jena, Germany) equipped with an Olympus OM-DE-M10 digital camera (Olympus, Tokyo, Japan). All images were captured at identical magnification settings. A micrometer calibration ruler (division value 0.01 mm) was photographed under the same optical conditions and used in subsequent calibration of measurements.

**Figure 3:**
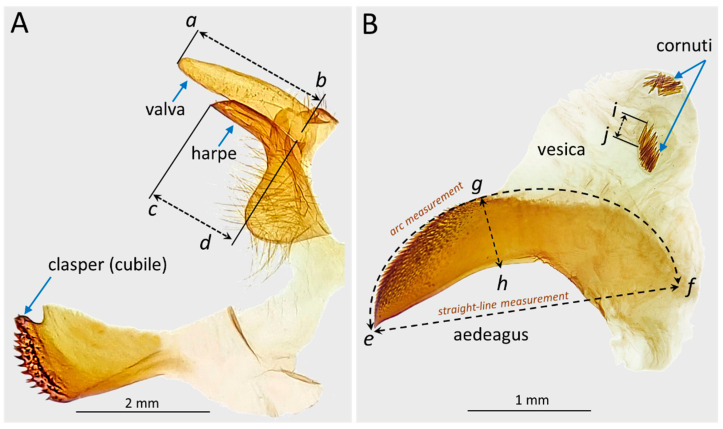
Male genitalia of *Dendrolimus* (using *D. sibiricus* as an example) and measured characters. (**A**)—peripheral structures (left half of the genital capsula): valva (*a*–*b*—measured length), harpe (*c*–*d*—measured length), clasper, or cubile (not studied as it bears no pronounced diagnostic characters); (**B**)—aedeagus: aedeagus (*e*–*f*—measured lengths in a straight line and in an arc, and *g*–*h*—width); cornuti on everted vesica (*i*–*j*—measured cornuti length). Photos: M.A. Ryazanova and N.I. Kirichenko.

Linear morphometric parameters were measured using CooRecorder 9.0 (Cybis Elektronik & Data, Saltsjöbaden, Sweden). For each specimen, the following structures were measured: valva length, harpe length, aedeagus length (in a straight line and in an arc, or a curved line), aedeagus width, and cornuti length (Figure 3).

The valva length was estimated as the distance from the apex to the base of valva (*a–b*) and the harpe length was measured as the distance from the harp apex to the point where the harpe meets the valva (*c–d*) (Figure 3A). The aedeagus length was estimated in a straight line, as a linear distance between the apex *e* and the most distant basal point *f*, and in an arc, as the distance measured along the dorsal contour of the aedeagus, between the same two endpoints (*e–f*) (Figure 3B). The measuring of the aedeagus length in an arc accounts for natural curvature and represents the functional length of the aedeagus. Aedeagus width was measured as a straight line drawn at a 90° angle between the most distant dorsal point *g* (where this point coincides with the beginning of the opening through which the vesica is everted) and the ventral point *h* (Figure 3B). The cornuti length was assessed as the linear distance from the apex *i* to the base of a single cornutus *j* (Figure 3B). For each specimen, ten cornuti were selected haphazardly for measurement (five from each symmetrically situated group on vesica, i.e., two cornuti in the marginal region and three cornuti in the center of the group) to take into account cornuti variability within vesica.

All measurements were taken in millimeters. Care was taken to position landmarks consistently across specimens to ensure measurement repeatability.

### 2.5. Data Analysis

For each measured genital structure, the mean value and standard deviation were calculated. Comparisons among taxa (*D. sibiricus*, *D. pini*, and their hybrids) were performed for the following morphometric characters: valva length, harpe length, aedeagus length (in a straight line and in an arc), aedeagus width, and cornuti length (Figure 3).

In addition to the values of measured genitalia structures, four genitalia indices were derived to capture proportional relationships among structures and to enhance diagnostic resolution: Harpe Length/Valva Length Index (HL/VL), Aedeagus Length/Aedeagus Width Index (AW/AL), Cornuti Length/Aedeagus Length Index (CL/AL), and Combined Genital Proportion Index (CGPI) (Table 1). In the indices where aedeagus length was involved (i.e., AW/AL, CL/AL, and CGPI), two calculations were performed using the aedeagus length measured in a straight line and in an arc to determine whether arced measurement enhanced differentiation capabilities among the studied taxa.

Statistical analyses were carried out using Statistica 14.0 software (TIBCO Software Inc., Palo Alto, CA, USA). Differences among taxa regarding both measured parameters and indices were evaluated through the nonparametric Mann–Whitney U test [33]. This approach was chosen given the small number of hybrid samples (four individuals compared to thirty each for *D. sibiricus* and *D. pini*). The relationships between genitalia structures were examined via linear regression analysis [34].

In order to assess the effectiveness of genitalia indices in distinguishing taxonomic groups, first, the discriminant analysis for the parental species (*D. sibiricus* and *D. pini*) was employed since their datasets satisfied the prerequisites necessary for this type of analysis [35]. The analysis was performed in two steps. At the first step, all indices were included to define the most powerful predictors. At the second step, the most power predictor was excluded, and for the remaining set of indices, the backward stepwise approach was employed because the analysis of the whole set did not allow us to define powerful predictors.

To classify all three taxa (including the hybrid group), the decision tree analysis utilizing the chi-square statistic as the split criterion was used [36]. Unlike discriminant analysis, it allowed for the analysis of imbalanced or smaller sample sizes, offering an efficient method for generating robust classification rules.

### 2.6. Mapping

The schematic map of *D. sibiricus* and *D. pini* ranges and their contact zone was created based on data from [3,7,14,15,17,18,19,20,21,23,24] and our present study. The presence of *D. pini* is not depicted on the map in China, as no precise regional data were found beyond a general mention of the country [18,21,37]. The presence of *D. pini* in the Republic of Sakha (Yakutia) is reported here for the first time based on the revision of genus *Dendrolimus* in the archival collection of ZIN RAS, i.e., the specimens from Olekminsk (11.07.1911, Kharitonov coll.). The points from where the insect specimens originated were indicated on the map in accordance with their sampling date and storing depository. The map was built using ArcGIS 9.3 (ESRI, Redlands, CA, USA) [31].

## 3. Results

### 3.1. Morphometric Values of Male Genital Structures

**Figure 4:**
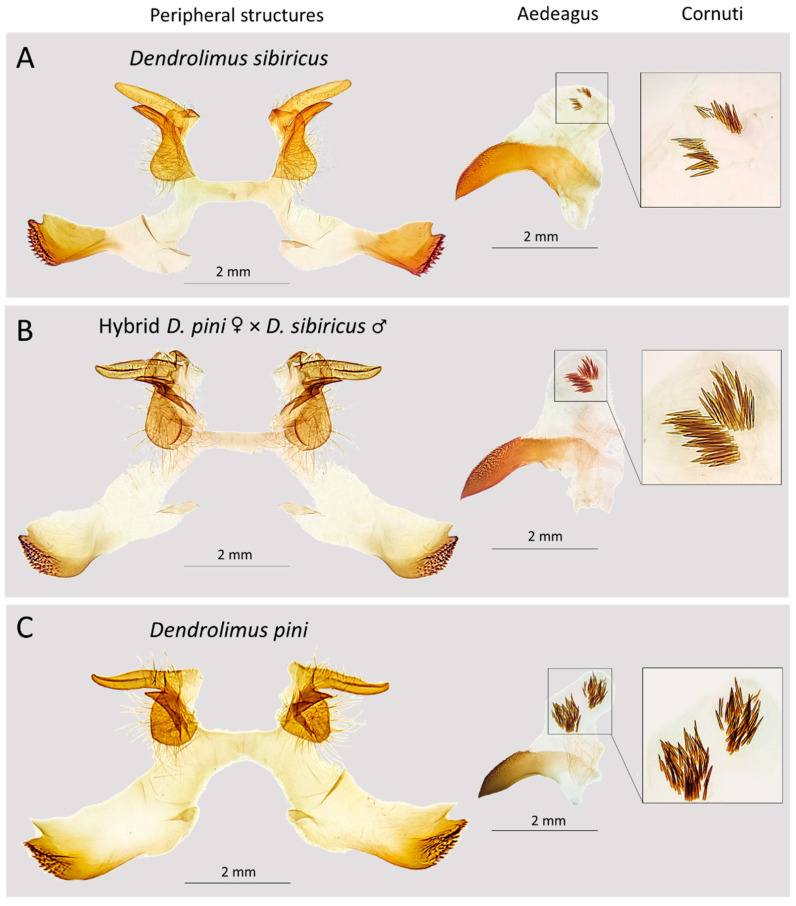
Comparative morphology of male genital structures in the studied representatives of *Dendrolimus*. (**A**)—*D. sibiricus*; (**B**)—hybrid *D. pini* ♀ × *D. sibiricus* ♂; (**C**)—*D. pini*. Photos: M.A. Ryazanova and N.I. Kirichenko.

**Figure 5:**
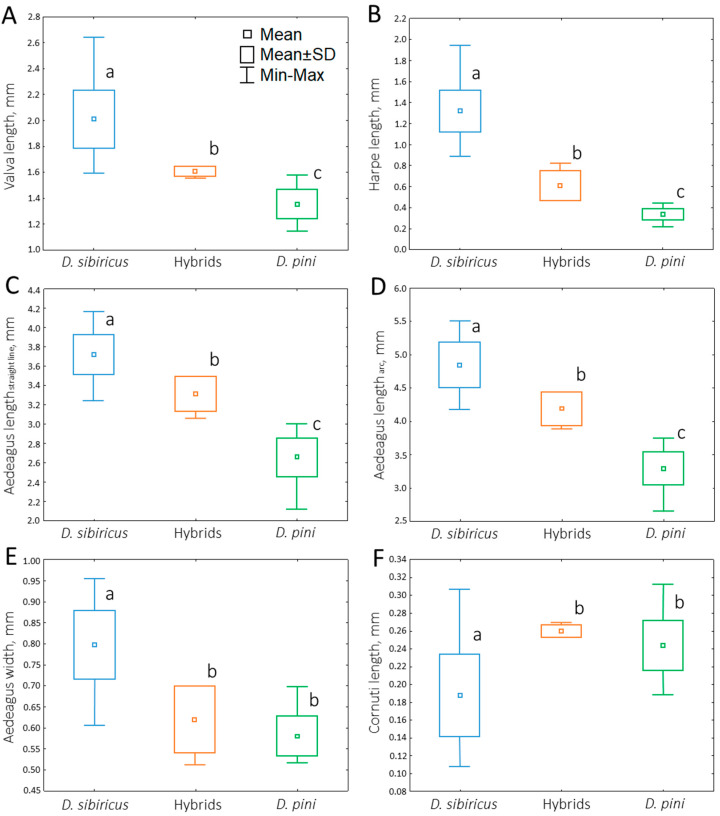
Morphometric values of male genital structures of *Dendrolimus sibiricus*, *D. pini* and their hybrids. (**A**)—valva length; (**B**)—harpe length; (**C**)—aedeagus length (measured in a straight line); (**D**)—aedeagus length (measured in an arc); (**E**)—aedeagus width; (**F**)—cornuti length. Central squares mark media, boxes indicate standard deviation, and whiskers show minimum and maximum values. Different letters next to the boxplots highlight significant differences between the boxplots, whereas the same letters indicate no difference (Mann–Whitney test; N*_D. sibiricus_* = 33, N*_D. pini_* = 33, and N_hybrids_ = 4; *p* < 0.01).

***Harpe and valva:*** The mean lengths of the harpe and valva differed significantly among the taxa (Figure 4 and Figure 5A,B).

In *D. sibiricus*, the valva and harpe were significantly longer than in *D. pini* (Mann–Whitney test: Z = 7.083 and *p* ≤ 0.01; Z = 7.084 and *p* ≤ 0.01, respectively). Mean valva and harpe lengths were 2.002 ± 0.226 mm and 1.312 ± 0.200 mm in *D. sibiricus* and 1.356 ± 0.112 mm and 0.339 ± 0.055 mm in *D. pini*. Hybrids occupied an intermediate position, with mean valva and harpe lengths of 1.609 ± 0.036 mm and 0.608 ± 0.142 mm, respectively. All pairwise comparisons revealed significant differences among taxa for both structures (*p* ≤ 0.01) (Figure 5A,B).

***Aedeagus length and width:*** Aedeagus length (both in a straight line and in an arc) decreased along the series *D. sibiricus* → hybrids → *D. pini* (Figure 5C,D). Mean straight-line lengths were 3.712 ± 0.204 mm (*D. sibiricus*), 3.315 ± 0.181 mm (hybrids), and 2.663 ± 0.200 mm (*D. pini*), whereas mean values of aedeagus length measured in an arc were 4.844 ± 0.334 (*D. sibiricus*) mm, 4.194 ± 0.250 mm (hybrids), and 3.295 ± 0.247 mm (*D. pini*). All comparisons within each taxon were significant for both measurements of aedeagus length: for *D. sibiricus* (Z = −6.976; *p* ≤ 0.01), hybrids (Z = −2.165; *p* ≤ 0.01), and *D. pini* (Z = −6.578; *p* ≤ 0.01). In all cases, the length of the aedeagus measured in an arc was about 27% greater than measured in a straight line.

Mean aedeagus width was 0.798 ± 0.080 mm (*D. sibiricus*), 0.619 ± 0.078 mm (hybrids), and 0.576 ± 0.052 mm (*D. pini*) (Figure 5E). *D. sibiricus* differed significantly from both *D. pini* (Z = 7.083; *p* ≤ 0.01) and hybrids (Z = 2.877; *p* ≤ 0.01), whereas *D. pini* and the hybrids showed no significant difference (Z = −1.070; *p* ≥ 0.1). As with other structures, *D. sibiricus* exhibited the largest values and *D. pini* the smallest, and the hybrids had an intermediate position (Figure 4 and Figure 5E).

***Cornuti length:*** Mean cornuti length was significantly smaller in *D. sibiricus* (0.188 ± 0.046 mm) compared to *D. pini* (0.243 ± 0.027 mm) (Z = −4.909; *p* ≤ 0.01) and hybrids (0.259 ± 0.01 mm) (Z = −2.812; *p* ≤ 0.01). No significant differences were found between *D. pini* and the hybrids (Z = −1.117; *p* ≥ 0.1) (Figure 4 and Figure 5F).

### 3.2. Relationship of Genital Structures

**Figure 6:**
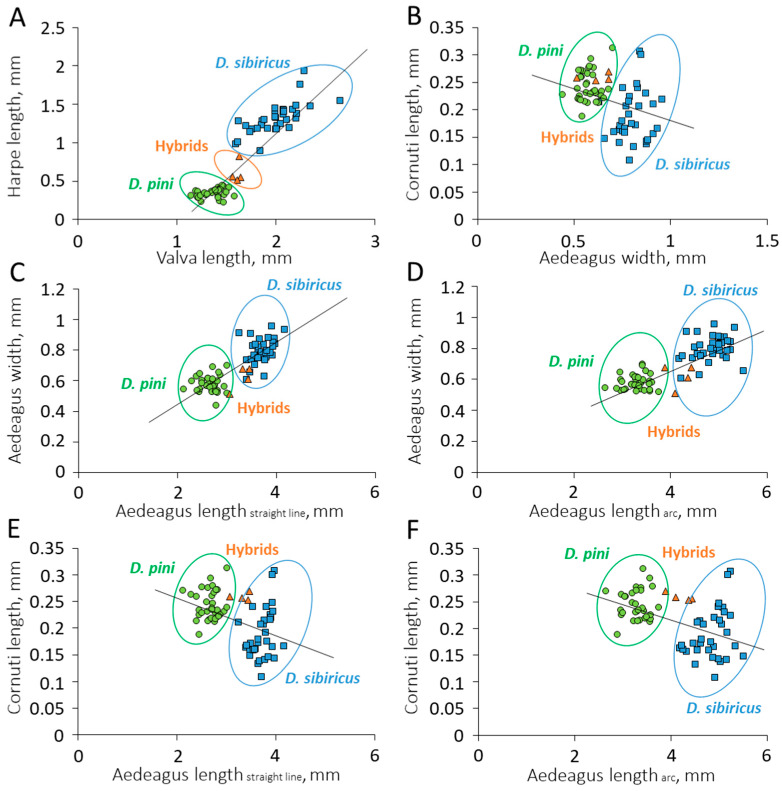
The relationship of genitalia structures in *Dendrolimus sibiricus*, *D. pini* and their hybrids and formed data clouds. (**A**)—harpe to valva length; (**B**)—cornuti length to aedeagus width; (**C**,**D**)—aedeagus width to aedeagus length (measured in a straight line and an arc respectively); (**E**,**F**)—cornuti length to aedeagus length (measured in a straight line and an arc respectively). The linear relationships are significant in all cases (see text).

A positive relationship was detected between harpe and valva lengths (y = 1.286x − 1.341, R^2^ = 0.861, and N = 70; *p* < 0.01). The ratio of harpe to valva length clearly separated the taxa, with species and hybrids forming distinct clusters (discriminant analysis: F = 85.250; *p* < 0.05) (Figure 6A).

Similarly, a positive relationship between aedeagus width and length was defined both for the length measured in a straight line (y = 0.193x + 0.065, R^2^ = 0.682, and N = 70; *p* < 0.01) and in an arc (y = 0.128x + 0.164, R^2^ = 0.670, and N = 70; *p* < 0.01). The ratio of aedeagus width to length in a straight line (Figure 6C) separated *D. sibiricus* and *D. pini* into two groups (F = 70.608; *p* ≤ 0.05); the same was true for width to length in an arc (F = 71.843; *p* ≤ 0.05) (Figure 6D). In both cases, hybrid specimens did not form a discrete cluster but instead entered the clusters of parental species.

Between cornuti length and aedeagus width, there was a negative linear relationship (y = −0.160x + 0.327, R^2^ = 0.196, and N = 70; *p* < 0.05) (Figure 6B). A negative relationship was also detected between cornuti length and aedeagus length measured in a straight line (y = −0.039x + 0.343, R^2^ = 0.217, and N = 70; *p* < 0.05) and cornuti length and aedeagus length measured in an arc (y = −0.027x + 0.329, R^2^ = 0.222, and N = 70; *p* < 0.05) (Figure 6E,F).

The ratio of cornuti length to aedeagus width separated *D. sibiricus* from *D. pini* (F = 39.669; *p* ≤ 0.05) (Figure 6B). The separation of these two species was also obvious when analyzing the ratio of cornuti length to aedeagus straight-line length (F = 73.689; *p* ≤ 0.05) (Figure 6E) and cornuti length to aedeagus arc length (F = 72.844; *p* ≤ 0.05) (Figure 6F). The hybrid specimens grouped either with one parental species (with *D. pini*, when analyzing cornuti length to aedeagus width) or with both parental species (cornuti length to aedeagus length measured in both variants).

In all cases, where aedeagus length was involved, the length measured in an arc provided similarly significant statistical values in both regression and discriminant analyses as the length measured in a straight line.

### 3.3. Male Genitalia Indices

**Figure 7:**
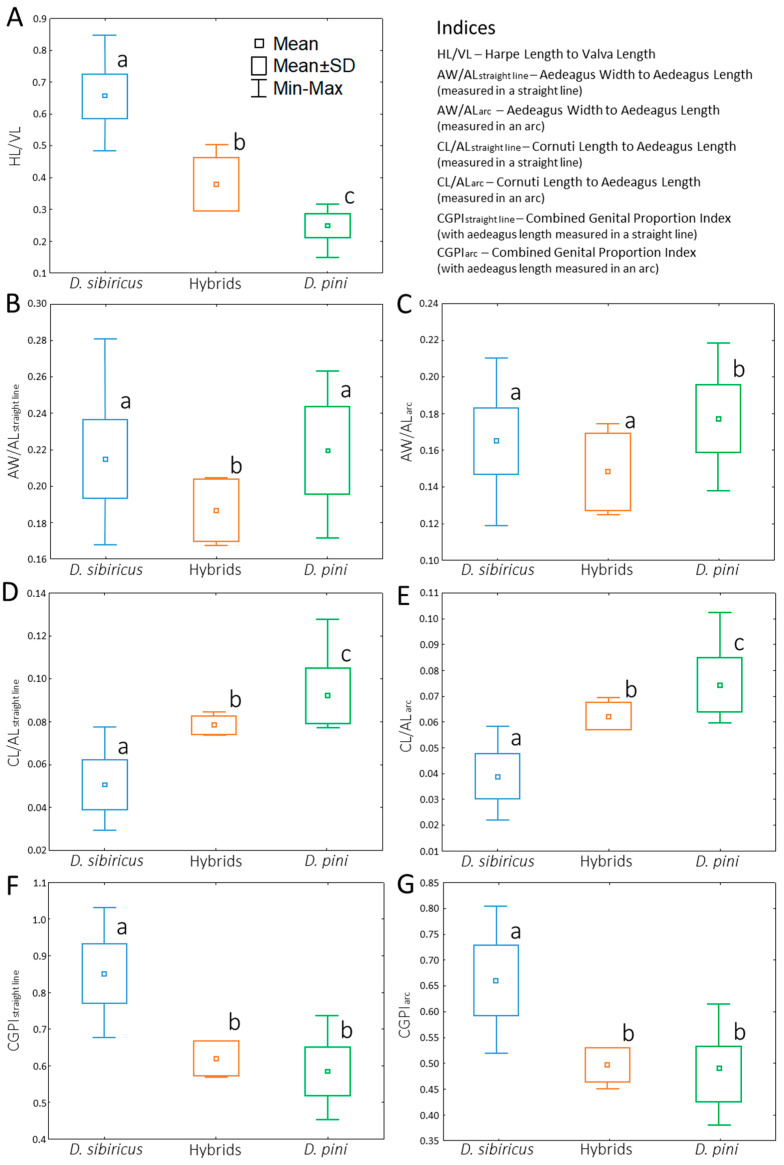
Indices of male genitalia structures assessed for *Dendrolimus sibiricus*, *D. pini*, and their hybrids. (**A**)—harpe length to valva length; (**B**,**C**)—aedeagus width to aedeagus length (the latter is in two measurement variants: in a straight line and an arc); (**D**,**E**)—cornuti length to aedeagus length (the latter in two measurement variants); (**F**,**G**)—combined genital proportion index (with aedeagus length measured in two variants). Central squares mark media, boxes indicate standard deviation, and whiskers show minimum and maximum values. Different letters next to the boxplots highlight a significant difference between the boxplots, whereas the same letters indicate no difference (Mann–Whitney test: N*_D. sibiricus_* = 33, N*_D. pini_* = 33, and N_hybrids_ = 4; *p* < 0.01).

The HL/VL Index differed significantly among all three taxa: *D. sibiricus* (0.656 ± 0.07), hybrids (0.378 ± 0.085), and *D. pini* (0.251 ± 0.038) (Mann–Whitney; Z*_D. sibiricus–D. pini_* = 7.083 and *p* < 0.01; Z*_D. sibiricus_*_–hybrids_ = –3.163 and *p* < 0.01; Z*_D. pini_*_–hybrids_ = 3.211 and *p* < 0.01) (Figure 7A).

The AW/AL Index was computed in two variants (with aedeagus length measured in a straight line and in an arc) produced inconsistent results. In the first case (AW/AL, straight line), *D. sibiricus* (0.216 ± 0.022) was indistinguishable from *D. pini* (0.218 ± 0.026) (Z = –0.497; *p* ≥ 0.1), whereas the hybrids exhibited a lower mean value differing from both parental species (Z*_D. sibiricus_*_–hybrids_ = –2.497 and Z*_D. pini_*_–hybrids_ = –2.259; *p* < 0.01) (Figure 7B). In the second case (AW/AL, arc), the difference was statistically significant between *D. sibiricus* (0.165 ± 0.018) and *D. pini* (0.177 ± 0.019) (Z = −2.577; *p* < 0.01) and between the hybrids (0.148 ± 0.021) and *D. pini* (Z = −2.274; *p* < 0.05) but not between the hybrids and *D. sibiricus* (Z = −1.546; *p* ≥ 0.1) (Figure 7C).

The CL/AL Index was computed in two variants for aedeagus length revealed clear distinctions among all three studied taxa (Figure 7D,E). In the first case (CL/AL, straight line), the mean values were as follows: 0.051 ± 0.012 (*D. sibiricus*), 0.080 ± 0.004 (hybrids), and 0.092 ± 0.013 (*D. pini*) (Z*_D. sibiricus–D. pini_* = 7.004, Z*_D. sibiricus_*_–hybrids_ = –3.106, and Z*_D. pini_*_–hybrids_ = –2.402; *p* < 0.01) (Figure 7D). In the second case (CL/AL, arc), the index showed following mean values: 0.039 ± 0.009 (*D. sibiricus*), 0.080 ± 0.004 (hybrids), and 0.092 ± 0.013 (*D. pini*) (Z*_D. sibiricus–D. pini_* = −6.977 and *p* < 0.01; Z*_D. sibiricus_*_–hybrids_ = 3.057 and *p* < 0.01; Z*_D. pini_*_–hybrids_ = −2.421 and *p* < 0.05) (Figure 7E).

The CGPI was also computed in two variants for aedeagus length distinguished between the parental species and between *D. sibiricus* and the hybrids but not between *D. pini* and the hybrids (Figure 7F,G). The mean values of CGPI_straight line_ were as follows: 0.852 ± 0.081 (*D. sibiricus*), 0.621 ± 0.048 (hybrids), and 0.586 ± 0.065 (*D. pini*) (Z*_D. sibiricus–D. pini_* = 6.979, Z*_D. sibiricus_*_–hybrids_ = –3.203, and *p* < 0.01; Z*_D. pini_*_—hybrids_ = 0.975 and *p* ≥ 0.1) (Figure 7F). In the case of CGPI_arc_, they were as follows: 0.662 ± 0.068 (*D. sibiricus*), 0.499 ± 0.033 (hybrids), and 0.480 ± 0.054 (*D. pini*) (Z*_D. sibiricus–D. pini_* = 6.733 and *p* < 0.01; Z*_D. sibiricus_*_–hybrids_ = −3.155 and *p* < 0.01; Z*_D. pini_*_–hybrids_ = 0.954) (Figure 7G).

### 3.4. Discriminant Analysis for D. sibiricus and D. pini

Discriminant analysis run for measured male genitalia structures of *D. sibiricus* and *D. pini* defined four parameters, which notably contributed to the species differentiation, with harpe length as the strongest predictor (Table 2).

Discriminant analysis conducted using all male genitalia indices of *D. sibiricus* and *D. pini* revealed a single dominant predictor—the HL/VL Index (Table 3, Model 1)—which played a major role in differentiating the two parental species.

Upon excluding the primary predictor (HL/VL Index) and applying a backward stepwise selection procedure to the remaining indices, four additional influential predictors were identified, ranked in descending order of importance: CGPI_arc_ → CL/AL_straight line_ → AW/AL_arc_ → AW/AL_straight line_ (Table 3, Model 2).

### 3.5. Decision Tree for Identifying D. sibiricus, D. pini and Their Hybrids

**Figure 8:**
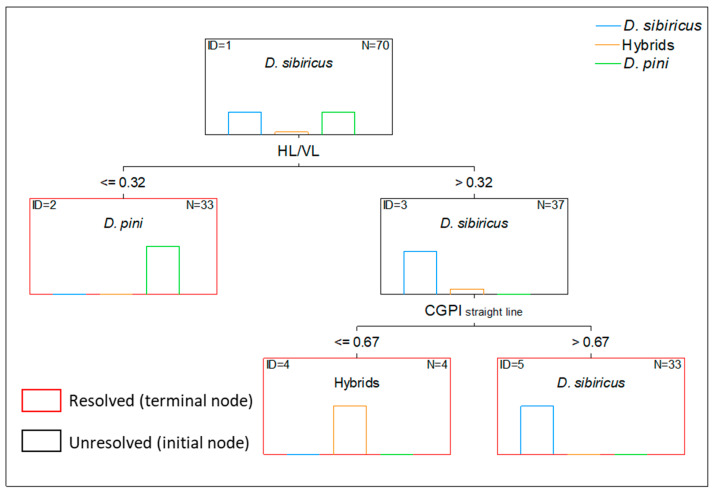
The decision trees based on male genitalia indices of *Dendrolimus sibiricus*, *D. pini* and their hybrids: HL/VL Index and CGPI_straight line_.

In the decision tree based on genitalia indices of all three taxa, two indices were the most relevant: HL/VL, which effectively separated *D. pini*, and CGPI, which separated hybrids from *D. sibiricus* (Figure 8).

Specifically, the specimens with a HL/VL Index ≤ 0.32 were assigned to *D. pini.* The remaining specimens were separated into *D. sibiricus* and hybrid groups based on the CGPI, with hybrids exhibiting lower values.

## 4. Discussion

In Lepidoptera, male genitalia structures are among the most evolutionarily important traits [38,39,40], serving as consistent species-level diagnostic characters [14,41]. However, in closely related species and in challenging cases involving hybrids, qualitative descriptions of genitalia alone may be insufficient for reliable species identification and delineating parental species and their hybrids. Therefore, quantitative measurements of genitalia structures can be essential for accurate diagnostics of closely related species and their hybrids. Indeed, quantitative analysis of male genital morphology has been proven to provide a more powerful and objective tool for species identification in Lepidoptera than subjective visual assessment, and, as such, morphometric approaches have successfully resolved closely related taxa in a number of moth and butterfly groups [41,42,43].

***Morphometric differentiation between D. sibiricus and D. pini:*** Our analysis demonstrates that male genital morphometry offers a statistically significant tool to distinguish *D. sibiricus* and *D. pini.* Morphometric values of examined structures—valva and harpe lengths, aedeagus length and width, and cornuti length—differ significantly between the two species, validating earlier qualitative descriptions [14] and providing the first quantitative basis for routine species diagnostics.

The length of the harpe showed the greatest proportional divergence, with *D. sibiricus* exhibiting markedly longer harpes than *D. pini*. Valva and aedeagus lengths also reliably distinguish the two species. Aedeagus length measured along both straight line and arc showed congruent species separation, suggesting that the simpler straight-line measurement may be sufficient for diagnostic purposes. Cornuti length displayed interspecific variability in studied species of *Dendrolimus*, a phenomenon also reported in other Lepidoptera [44]. Nevertheless, cornuti length, despite significant interspecific differences, can vary within one species and thus should be used cautiously, preferably in combination with other genital characters.

***Hybrid morphology and implications for species boundaries:*** Hybrid males, which resulted from crossing *D. pini* females and *D. sibiricus* males, showed intermediate values for some genitalic characters (in particular harpe and valva length), supporting the hypothesis of additive inheritance. This hypothesis suggests that hybrid phenotypes represent the averaged contributions of both parents, without dominance of either parental trait [45]. This pattern is consistent with observations from other Lepidoptera species, where hybrids show intermediate, approximately additive phenotypes [46,47,48,49]. In addition, cases of one-way introgression and maternally biased genetic contributions documented in hybrid zones of some Lepidoptera support the possibility of maternal influence or asymmetric dominance in specific traits [49]. This seems to be true for *Dendrolimus* species.

Although the number of hybrid specimens was small (i.e., four specimens in total), the consistent intermediate morphology and size of structures indicate that hybrid individuals should, in principle, be morphometrically detectable in natural populations, particularly through harpe and valva lengths.

***Diagnostic indices and their applied value:*** Several studies have demonstrated that morphometric ratios of genitalia elements, including length of valva, aedeagus and associated structures, indicate consistent and diagnostic species-level differences [41,42,43]. In butterflies, geometric and linear morphometrics of genitalia have successfully distinguished cryptic or near-cryptic species [50,51], whereas in moths, ratio-based indices have been shown to outperform subjective qualitative assessment in both accuracy and repeatability [42].

Our results demonstrate comparable utility of genital indices for *Dendrolimus* species. The provided values of the indices serve as reliable reference data to distinguish *D. sibiricus*, *D. pini*, and their hybrids (Table 4).

The diagnostic indices proposed here—the Harpe Length/Valva Length Index (HL/VL) and Cornuti Length/Aedeagus Length Index (CL/AL)—are highly informative as they differentiate between all three taxa. The Combined Genital Proportion Index (CGPI), which takes into account the genitalic parameters of different diagnostic values, is efficient for parental species determination (independently of whether the aedeagus length was measured on a straight line or in an arc) and for distinguishing between *D. sibiricus* and the hybrids but not between *D. pini* and the hybrids (Table 4). The other index, Aedeagus Width/Aedeagus Length, is the least informative: depending on how the aedeagus length is measured, it can provide satisfactory differentiation between *D. sibiricus* and *D. pini*, and *D. pini* and hybrids (when measured in an arc), but it does not allow for differentiation between *D. sibiricus* and *D. pini* and only distinguishes the hybrids from the parental species (when measured in a straight line) (Table 4).

Among these, the HL/VL Index showed particularly strong discriminatory power and corresponds closely to long-recognized qualitative differences in harpe morphology between *D. sibiricus* and *D. pini* [14]. Because the harpe and valva are structures that are relatively easy to measure and show limited within-specimen deformation during slide preparation, the HL/VL Index is especially suitable for rapid diagnostics of *D. sibiricus*, *D. pini* and their hybrids.

The Cornuti Length/Aedeagus Length Index (CL/AL) proves highly informative; however, when this index is used in discriminant analysis, it shows much less predictive power for taxa discrimination compared with the HL/VL Index. Moreover, the estimation process of the CL/AL Index is significantly more complex, requiring advanced preparatory techniques. Specifically, it involves carefully everting the vesica from the aedeagus and precisely measuring the lengths of at least ten cornuti. Additionally, complications arise when cornuti are absent due to copulation events occurring prior to male dissection. Indeed, during copulation, moth males often transfer cornuti into the female’s genital tract; thus, they can be missing in vesica [52,53].

The measurement of aedeagus length along an arc modestly improves discrimination in relevant indices compared with straight-line measures. Nevertheless, the CL/AL Index using the straight-line length effectively distinguishes both parental species and the hybrids, while the CGPI separates *D. sibiricus* from *D. pini* and hybrids. Therefore, routine taxon identification can reliably rely on simple straight-line measurements.

Multivariate analyses further reinforce the utility of the indices. Decision tree analysis produced clear species separation, with high classification accuracy across all taxa (*D. sibiricus*, *D. pini* and hybrids). Decision trees identified harpe length and aedeagus length as the most informative variables—traits that are straightforward to record and display stable interspecific differences even in the presence of hybrid individuals. Such findings are consistent with other Lepidopteran groups, where decision tree or linear-discriminant frameworks based on genitalia ratios have successfully separated morphologically similar species or hybrid complexes, e.g., hybrids of *Papilio* species [54] or *Leptidea* species [50].

Altogether, these results highlight the value of genitalia indices for *Dendrolimus* species and support the broader conclusion, demonstrated across Lepidoptera, that quantitative genitalic metrics offer a powerful and objective tool for species identification, supplementing traditional taxonomy based on qualitative descriptions.

***Implications for monitoring:*** Given the potential westward expansion of *D. sibiricus*, its partial sympatry with *D. pini*, and the demonstrated capacity of these species to hybridize under laboratory conditions, the development of reliable and operationally simple diagnostic tools is essential for quarantine and forest health monitoring programs. Hybrids could complicate surveillance efforts by blurring morphological boundaries and confounding pheromone-based detection, especially because *D. pini* males are attracted to the synthetic sex pheromone of *D. sibiricus* [15,27]. If hybrids combine adaptive traits of both species—such as the climatic tolerance and phenological flexibility of *D. pini* with the high outbreak potential and broader host range of *D. sibiricus*—they could present an unrecognized threat to European conifer forests [24,55].

***Study advances and limitations:*** This study provides the first quantitative and statistically supported framework for distinguishing *D. sibiricus*, *D. pini*, and their hybrids based on male genital morphometry, demonstrating clear diagnostic value in several genitalic structures and introducing practical ratio-based indices with high discriminatory power. These advances substantially improve the accuracy and repeatability of morphological identification compared with traditional qualitative taxonomy and offer immediate utility for monitoring and quarantine programs.

However, several limitations should be noted. The number of hybrid specimens analyzed in our study was small (only four specimens), reducing the statistical power to assess the full range of hybrid variation. Moreover, only one hybrid variant— *D. pini* (♀) × *D. sibiricus* (♂)—was available for examination; the reciprocal hybrid combination, i.e., *D. sibiricus* (♀) × *D. pini* (♂), was missing but it could exhibit different morphological outcomes. This constraint limits the generality of conclusions regarding hybrid diagnostic characters. Experimental studies with crosses of *D. sibiricus* and *D. pini*, the obtainment of larger sampling sets for robust statistical analysis and the inclusion of the missing hybrid variant would be desirable.

Finally, morphometric data alone may not fully resolve species boundaries or detect introgression in sympatric areas of the two *Dendrolimus* species. Integrating molecular diagnostics, including DNA barcoding and genomic tools, will therefore be essential for validating morphological identifications, detecting hybridization in natural populations, and refining our understanding of species limits and invasion risks within the *Dendrolimus* species complex.

## 5. Conclusions

Morphometric values of male genitalia and estimated indices provide a reliable, reproducible, and practical framework to distinguish *D. sibiricus*, *D. pini*, and their hybrids. The major points from the present study can be summarized as follows:

1. Clear morphometric separation between *D. sibiricus* and *D. pini.* All examined genitalia structures—valva length, harpe length, aedeagus length and width, and cornuti length—differed statistically between the two species. These results quantitatively confirm earlier qualitative descriptions and firmly establish male genitalia morphometry as a robust diagnostic tool for distinguishing the two major forest pests.

2. Harpe and valva lengths are the most robust diagnostic traits. Among all the structures measured, the harpe and valva exhibited the greatest proportional divergence and the strongest discriminatory power. These traits are consistently stable across individuals, are easy to measure, and reliably separate *D. sibiricus* from *D. pini*, making them especially suitable for routine monitoring and quarantine diagnostics.

3. Hybrid males exhibit intermediate morphology consistent with additive inheritance. Hybrids derived from *D. pini* (♀) × *D. sibiricus* (♂) crosses showed intermediate values for valva and harpe length, supporting the hypothesis that these genitalic traits are inherited in an approximately additive manner. These two measurements were the only structures that consistently differentiated hybrids from both parental species.

4. Aedeagus width and cornuti length are less informative for hybrid detection. Although these traits differed significantly between *D. sibiricus* and *D. pini*, neither aedeagus width nor cornuti length reliably separated hybrids from *D. pini*, largely due to higher within-species variability and overlap. These characters should therefore not be used alone for hybrid identification.

5. Genitalia indices and multivariate approaches enhance diagnostic accuracy. Harpe Length/Valva Length Index (HL/VL) proved highly effective to discriminate *D. sibiricus* from *D. pini* and *D. pini* from hybrids, whereas Combined Genital Proportion Index (CGPI) to discriminate *D. sibiricus* from hybrids. Discriminant analysis and decision tree models further confirmed that these indices provide strong classification accuracy and can be used for rapid diagnostics.

6. Integrated morphological and molecular approaches are essential for future monitoring. While genital morphometry offers a powerful first-line identification tool, molecular markers will be required to clarify species boundaries and detect hybridization in natural populations. This is particularly important given the westward expansion of *D. sibiricus*, pheromone-trap cross-attraction with *D. pini*, and the potential ecological risks posed by hybrids of these two forest pest species.

## Figures and Tables

**Table 1:** Indices of male genitalia based on diagnostic characters and their definitions.

Indices *	Definition	What It Captures; Why Helpful
Harpe Length/Valva Length Index (HL/VL)	harpe length to valva length	captures relative size of harpe vs. valva; helpful if one species has longer harpe than another
Aedeagus Width/Aedeagus Length Index (AW/AL)	aedeagus width to aedeagus length	captures relative size of aedeagus; helpful if one species has wider aedeagus than another
Cornuti Length/Aedeagus Length Index (CL/AL)	cornuti length to aedeagus length	captures relative size of cornuti vs. aedeagus; helpful if one species has wider aedeagus than another
Combined Genital Proportion Index (CGPI)	(valva length + harpe length) to (aedeagus length + cornuti length)	captures relative size of multiple structures; helpful if one species has larger multiple structures than another

* Three indices (AW/AL, CL/AL, and CGPI) were calculated in two variants involving aedeagus length measured in a straight line and an arc.

**Table 2:** Discriminant model using male genitalia parameters of *D. sibiricus* and *D. pini* as predictors.

Genital Structures	F-Remove	*p*-Value
Harpe length *	35.882	≤0.01
Cornuti length *	12.370	≤0.01
Valva length *	4.557	≤0.05
Aedeagus length, measured in an arc *	4.265	≤0.05
Aedeagus width	3.510	≥0.05
Aedeagus length, measured in straight line	1.164	≥0.1

* The parameters contributed to the species differentiation notably.

**Table 3:** Discriminant models using male genitalia indices of *D. sibiricus* and *D. pini* as predictors.

Indices of Male Genitalia Structures	F-Remove	*p*-Value
Model 1 (includes all indices)		
HL/VL *	87.540	≤0.01
CL/AL_straight line_	1.947	≥0.1
AW/AL_straight line_	1.711	≥0.1
AW/AL_arc_	1.588	≥0.1
CL/AL_arc_	1.311	≥0.1
CGPI_arc_	0.904	≥0.1
CGPI_straight line_	0.673	≥0.1
Model 2 (excludes HL/VL Index)		
CGPI_arc_ *	44.061	≤0.01
CL/AL_straight line_ *	41.035	≤0.01
AW/AL_arc_ *	17.869	≤0.01
AW/AL_straight line_ *	13.705	≤0.01

* The parameters contributed to the species differentiation notably.

**Table 4:** Reference values of male genitalia indices for differentiation between *Dendrolimus sibiricus*, *D. pini* and their hybrids.

Indices	Taxa	Reference Index Values		
		Mean ± SD *	Min	Max
Highly informative indices (enabling differentiation between *D. sibiricus*, *D. pini* and their hybrids)				
HL/VL	*D. sibiricus*	0.656 ± 0.069 a	0.484	0.847
	Hybrids	0.378 ± 0.085 b	0.317	0.503
	*D. pini*	0.251 ± 0.038 c	0.149	0.316
CL/AL_straight-line_	*D. sibiricus*	0.051 ± 0.012 a	0.029	0.077
	Hybrids	0.078 ± 0.004 b	0.073	0.085
	*D. pini*	0.092 ± 0.013 c	0.077	0.128
CL/AL_arc_	*D. sibiricus*	0.039 ± 0.009 a	0.022	0.059
	Hybrids	0.062 ± 0.005 b	0.058	0.069
	*D. pini*	0.074 ± 0.010 c	0.060	0.102
Informative indices (enabling differentiation between *D. sibiricus*, *D. pini*, and between only one of the parental species and their hybrids)				
CGPI_straight-line_	*D. sibiricus*	0.852 ± 0.081 a	0.677	1.031
	Hybrids	0.621 ± 0.048 b	0.568	0.664
	*D. pini*	0.586 ± 0.065 b	0.453	0.736
CGPI_arc_	*D. sibiricus*	0.662 ± 0.068 a	0.522	0.808
	Hybrids	0.499 ± 0.033 b	0.452	0.531
	*D. pini*	0.480 ± 0.054 b	0.381	0.617
AW/AL_arc_	*D. sibiricus*	0.165 ± 0.018 a	0.120	0.210
	Hybrids	0.148 ± 0.021 a	0.125	0.174
	*D. pini*	0.177 ± 0.019 b	0.138	0.218
The least informative index (enabling differentiation of hybrids from their parental species but not the parental species themselves)				
AW/AL_straight-line_	*D. sibiricus*	0.215 ± 0.022 a	0.168	0.280
	Hybrids	0.186 ± 0.017 b	0.167	0.205
	*D. pini*	0.218 ± 0.026 a	0.159	0.263

* Within each index, the values indicated by different letters are significantly different (Mann–Whitney test: N*_D. sibiricus_* = 33, N*_D. pini_* = 33, and N_hybrids_ = 4; *p* < 0.01).

## Data Availability

The raw data supporting the conclusions of this article will be made available by the authors on request.

## Supplementary Materials

The following supporting information can be downloaded at: https://www.mdpi.com/article/10.3390/life16030398/s1, Table S1: The specimens of *Dendrolimus sibiricus*, *D. pini* and their hybrids involved in the study: their origin and depositories.
