# Supplementary material for: The Morphometry of Male Genitalia as a Reliable Tool for Identifying Forest Pests Dendrolimus sibiricus, D. pini (Lepidoptera: Lasiocampidae), and Their Hybrids in Eurasia
Source: Life (Basel). 2026 Mar 1;16(3):398. doi: 10.3390/life16030398 (PMC13027563; doi:10.3390/life16030398)
Supplement: Supplementary file 1 [file life-16-00398-s001.zip › life-4166512-supplementary.pdf]

TITLE: The Morphometry of Male Genitalia as a Reliable Tool for Identifying Forest Pests, *Dendrolimus sibiricus, D. pini* (Lepidoptera: Lasiocampidae), and Their Hybrids in Eurasia

AUTHORS: Ryazanova M.A., Ageev A.A., Sinev S.Yu., Matov A.Yu., Gomboc S., Kovalenko M.G., Akulov E.N., Demidko D.A., Musolin D.L., Kirichenko N.I.

TABLE S1. The specimens of *Dendrolimus sibiricus, D. pini* and their hybrids involved to the study: their origin and depositaries.

No.	Country	Region[1], place (where known)	Collection date	Collector[2]	Sampling method[3]	Deposita-ries[4]
			Dendrolimus sibiricus			
1	Russia	Amur Reg., Ulunga river	1910	Mishin, Verkhovskaya	unknown	ZIN
2	Russia	Irkutsk Reg., Slyudyansky district, Kultuk village	1927	Froloff D.	unknown	ZIN
3	Russia	Irkutsk Reg., Slyudyansky district, Kultuk village	10.07.1927	Froloff D.	unknown	ZIN
4	Russia	Khakassia Rep., Shirinsky district	15.07.2024	Demidko D.A.	PhT	SCDD
5	Russia	Khakassia Rep. Shirinsky district	14.07.2009	Akulov E.N.	PhT	VNIIKR
6	Russia	Krasnoyarsk Ter., Kansk	20.07.2024	Akulov E.N.	PhT	VNIIKR
7	Russia	Magadan Reg., Tenkinsky district, Sibik-Tyellakh	06.07.1877	Zhiltsova L.	unknown	ZIN
8	Russia	Perm Ter., Kungur	15.07.1955	Zinoviev	unknown	ZIN
9	Russia	Primorsky Ter., Vladivostok, Sedanka district	25.07.1918	Krieger-Voynovsky	unknown	ZIN
10	Russia	Primorsky Ter.	1894	Hristinich	unknown	ZIN
11	Russia	Primorsky Ter., Suputinka River	06.07.1836	unknown	unknown	ZIN
12	Russia	Tomsk Reg.	09.08.1925	unknown	unknown	ZIN
13	Russia	Yakutia Rep., Olekminsk	15.07.1911	Kharitonov	unknown	ZIN
14	Russia	Yakutia Rep., Olekminsk	11.07.1911	Kharitonov	unknown	ZIN
15	Russia	Yakutia Rep., Olekminsk	11.07.1911	Kharitonov	unknown	ZIN
16	Russia	Yakutia Rep., Olekminsk	11.07.1916	Kharitonov	unknown	ZIN
17	Russia	Yakutia Rep., Olekminsk	11.07.1916	Kharitonov	unknown	ZIN
18	Russia	Yakutia Rep., Olekminsk	11.07.1916	Kharitonov	unknown	ZIN
19	Russia	Yakutia Rep., Olekminsk	11.07.1916	Kharitonov	unknown	ZIN

No.	Country	Region[1], place (where known)	Collection date	Collector[2]	Sampling method[3]	Deposita-ries[4]
20	Russia	Yakutia Rep., Olekminsk	11.07.1916	Kharitonov	unknown	ZIN
21	Russia	Zabaikalsky Ter.	1937	Teploukhov	unknown	ZIN
22	Russia	Zabaikalsky Ter.	1937	Teploukhov	unknown	ZIN
23	Russia	Zabaikalsky Ter.	1937	Teploukhov	unknown	ZIN
24	Russia	Zabaikalsky Ter.	1937	Teploukhov	unknown	ZIN
25	Russia	Zabaikalsky Ter.	1937	Teploukhov	unknown	ZIN
26	Russia	Zabaikalsky Ter.	1937	Teploukhov	unknown	ZIN
27	Russia	Zabaikalsky Ter.	1937	Teploukhov	unknown	ZIN
28	Russia	Primorsky Terr., Suchansky mine	19.07.1934	Palshkov	unknown	ZIN
29	Russia	Siberia	1910	Meinhardt A.	unknown	ZIN
30	Russia	Primorsky Terr., Suchansky mine	19.07.1934	Palshkov	unknown	ZIN
31	China	Beijing, Taihang Mts, Xiao Longmeng, Nat. Forest Park	08.07.2006	Krivokhatsky V.	unknown	ZIN
32	Kazakhstan	Semipalatinsk	06.05.1905	Lonshchakov	unknown	ZIN
33	Kazakhstan	Semipalatinsk	06.05.1905	Lonshchakov	unknown	ZIN
Dendrolimus pini						
34	Russia	Ivanovo Reg., Yuzhsky district	17.09.2024	unknown	PhT	VNIIKR
35	Russia	Ivanovo Reg., Yuzhsky district	18.09.2024	unknown	PhT	VNIIKR
36	Russia	Leningrad Reg., Vyborg province, Perkyavsk	23.06.1906	Diakonova	unknown	ZIN
37	Russia	Leningrad Reg., Saint Petersburg	03.06.1908	no data	unknown	ZIN
38	Russia	Leningrad Reg., Tikhvin district, Tikhvin city	23.07.2009	Matov A.	unknown	ZIN
39	Russia	Moscow Reg., Bykovo	28.06.2007	Ponomarev V.	unknown	VNIIKR
40	Russia	Moscow Reg., Bykovo	25.06.2007	Ponomarev V.	unknown	VNIIKR
41	Russia	Moscow Reg., Bykovo	22.06.2007	Ponomarev V.	unknown	VNIIKR
42	Russia	Perm Ter., Perm, Nizhnyaya Kurya	05.07.1918	Dyakonov A.	unknown	ZIN
43	Russia	Buryatia Rep., Ulan-Ude	27.06.2024	Kovalenko M.G.	PhT	VNIIKR
44	Russia	Buryatia Rep., Ulan-Ude	04.07.2024	Kovalenko M.G.	PhT	VNIIKR
45	Russia	Buryatia Rep., Ulan-Ude	04.07.2024	Kovalenko M.G.	PhT	VNIIKR
46	Russia	Buryatia Rep.,	04.07.2024	Kovalenko M.G.	PhT	VNIIKR

No.	Country	Region[1], place (where known)	Collection date	Collector[2]	Sampling method[3]	Deposita-ries[4]
		Ulan-Ude				
47	Russia	Buryatia Rep., Ulan-Ude	04.07.2024	Kovalenko M.G.	PhT	VNIIKR
48	Russia	Buryatia Rep., Ulan-Ude	04.07.2024	Kovalenko M.G.	PhT	VNIIKR
49	Russia	Yakutia Rep., Olekminsk	11.07.1911	Kharitonov	unknown	ZIN
50	Russia	Tula Reg., Aleksin	15.07.1902	Kele	unknown	ZIN
51	Russia	Ulyanovsk Reg., Inzensky district	01.08.2017	Nedoshivina S.V.	unknown	ZIN
52	Abkhazia	Gulripshsky district, Chkhalta village	23.06.1982	Zagulyaev A.K.	unknown	ZIN
53	Croatia	Rogač	20.09.2024	Gomboc S.	L	SCSG
54	Croatia	Rogač	20.09.2024	Gomboc S.	L	SCSG
55	Croatia	Rogač	20.09.2024	Gomboc S.	L	SCSG
56	Croatia	Rogač	20.09.2024	Gomboc S.	L	SCSG
57	Croatia	Rogač	20.09.2024	Gomboc S.	L	SCSG
58	Croatia	Rogač	20.09.2024	Gomboc S.	L	SCSG
59	Croatia	Rogač	20.09.2024	Gomboc S.	L	SCSG
60	Croatia	Rogač	20.09.2024	Gomboc S.	L	SCSG
61	Croatia	Rogač	20.09.2024	Gomboc S.	L	SCSG
62	Croatia	Rogač	20.09.2024	Gomboc S.	L	SCSG
63	Georgia	Manglisi	01.07.1971	Zagulyaev A.K.	unknown	ZIN
64	Slovenia	Gorenjska, Brdo pri Kranju	23.09.2003	Gomboc S.	L	SCSG
65	Slovenia	Koritnice, Notranjska, Bac	07.07.2018	Gomboc S.	L	SCSG
66	Ukraine	Poltava	07.07.1928	Miljanovskij E.	unknown	ZIN
**Hybrids: *D. sibiricus* (male) × *D. pini* (female)**						
67	Russia	no data	1956	Kuznetzov V.I.	C	ZIN
68	Russia	no data	1956	Kuznetzov V.I.	C	ZIN
69	Russia	no data	1956	Kuznetzov V.I.	C	ZIN
70	Russia	no data	1956	Kuznetzov V.I.	C	ZIN

[1]Region: Ter. – Territory (syn. Krai), Rep. – Republic, Reg. – Region (syn. Oblast).

[2]Collector: the surnames and names are given according to the data provided on the labels attached to the insect specimens; in case the name and the second name of collectors were not indicated on the labels, only surnames are listed here.

[3]Sampling method: PhT – attracted to the pheromone trap with synthetic sex pheromone of *D. sibiricus;* L - attracted to the light; C – obtained by crossing *D. sibiricus* male with *D. pini* female indoor.

[4]Depositaries: ZIN – Zoological Institute of the Russian Academy of Sciences, St. Petersburg; VNIIKR – All-Russian Plant Quarantine Center, Bykovo, Moscow Reg.; SCSG – Scientific collection of Stanislav Gomboc; SCDD – Scientific collection of Denis A. Demidko.
